# Case of Successful Exhibition of the Ergot of Rye in Lingering Labour

**Published:** 1827-09

**Authors:** Charles Clay

**Affiliations:** Member of the Royal College of Surgeons, Edinburgh.


*Case of successful Exhibition of the Ergot of Rye in lingering Labour.* By CHARLES CLAY, Esq. Member of the Royal College of Surgeons, Edinburgh.

HOWEVER strong a claim any new remedy may have to public attention, yet the great exaggeration that generally attends the first reports of the efficacy of such remedies often leads medical men to doubt the truth of the reports regarding them, and by these means such remedies, however valuable they may be, are a long time before they get generally admitted into practice, and submitted to fair and impartial trials. Indeed, I may add that many remedies, during this journey to public confidence, sink into oblivion, and are seldom heard of again. There are yet other causes that operate strongly against the introduction of new improvements:—first, the surgeons of the old school are often so partial to remedies they have been in the habit of using for a number of years, that new discoveries are by them often treated with contempt; secondly, the extravagant charges which chemists and druggists exact for any new medicine often excludes the substance from that general circulation which it requires to establish its merits. Of late a number of different objects have been offered to general notice, and a number of cures, bordering on the miraculous, have been reported, to bring some favourite object into notice. The following case, however, will tend to show the effects of one of the newly-discovered articles of the materia medica.

Mrs. Darwell, of Ashton-under-Lyne, sent for me on the 2d of June, 1826, being then in labour of her fifth child. I had attended her on a former labour, which was a very tedious one, and, from the state of the present symptoms, I feared another equally lingering. I found her in a very emaciated state; the pulse slow and weak; some cough, expectoration of pus; slight diarrhœa; dim eye; the tongue of a brown tinge in the middle, dry and cracked; she had not had any sleep for three or four nights. She considered herself at her full time, and had felt pains for two or three days. The pains at first were powerful, but they gradually diminished till she could scarcely discern them. She thought herself sinking fast, and eagerly wished for assistance. As the os uteri was fully dilated, I concluded the labour would terminate well, if rest was procured: I therefore ordered an opiate. She slept for some hours: the pains returned, but without any effect on the child, and, after the pains had been acting about one hour and a half, they began to decline again. I now determined to give the ergot of rye, and made an infusion of it in the following manner:

℞. Ergot ℈viij.; Aquæ Ferventis ℥ vj. Pour the water on the ergot after it has been well bruised in a mortar, and let it stand for fifteen minutes; then pour off the water, and bruise the ergot again; afterwards put the same liquid to it, and let it stand for ten minutes more.

Of this infusion I gave her three ounces. In a few minutes the pains were more powerful; but I considered them not sufficiently strong to expel the child, and therefore gave her the remaining three ounces. The pains soon became surprisingly altered, and in twenty minutes after the second dose, and thirty-five minutes after the first, the child was born. It was a large one, and perfectly healthy. From the powerful excitement produced by the exhibition of the ergot, I was afraid that the uterine action would remain after the child was born; but in this I was mistaken: for no sooner were the child and placenta expelled, than the excitement immediately ceased. The woman and child did well.

I am positive that in this case the whole success depended on the ergot. I have conversed with different medical men on the use of this medicine, many of whom doubt its action; but, on particular inquiry, I have generally found that they have given the ergot in powder, and in small doses,—seldom more than a drachm. The ergot should only be used in the last extremity, when the pains are completely leaving the patient, and when there is no prospect of a spontaneous re-action. It should never be given in less than eight scruples for two doses, infused in six ounces of boiling water: no more than fifteen or twenty minutes should elapse between the first and second doses. When given in this manner, success will attend it, in cases proper for its administration.

*Ashton-under-Lyne; August* 1827.

